# Mesoporous Silicas of Well-Organized Structure: Synthesis, Characterization, and Investigation of Physical Processes Occurring in Confined Pore Spaces

**DOI:** 10.3390/ijms26189255

**Published:** 2025-09-22

**Authors:** Magdalena Blachnio, Malgorzata Zienkiewicz-Strzalka, Anna Derylo-Marczewska

**Affiliations:** Department of Physical Chemistry, Institute of Chemical Sciences, Maria Curie-Sklodowska University, Maria Curie-Sklodowska Square 3, 20-031 Lublin, Poland; malgorzata.zienkiewicz-strzalka@mail.umcs.pl (M.Z.-S.); anna.derylo-marczewska@mail.umcs.pl (A.D.-M.)

**Keywords:** mesoporous silica, mesostructured cellular foams, structure-directing agents, Pluronic, dye adsorption, adsorption kinetics, adsorption equilibrium

## Abstract

Mesoporous silica materials with well-organized architectures were synthesized using a series of Pluronic PE-type triblock copolymers (PE6800, PE9200, PE9400, PE10500) as structure-directing agents under acidic conditions. The study aimed to elucidate the impact of synthesis parameters—copolymer type, presence of a swelling agent, 1,3,5-trimethylbenzene, aging temperature, and silica precursor—on the structural, textural, and functional properties of the resulting mesocellular foam materials. Characterization by Nitrogen Adsorption/Desorption, Transmission Electron Microscopy, X-ray Diffraction, and Small-angle X-ray Scattering revealed that structural ordering and pore morphology are significantly influenced by the EO/PO ratio of the copolymers and the use of the expander. Materials synthesized with PE9400 and PE10500 in the presence of a swelling agent exhibited highly uniform bottle-shaped mesopores with increased surface area and pore volume. Thermal behavior studied via Differential Scanning Calorimetry indicated a correlation between pore size and melting point depression of confined water, consistent with the Gibbs–Thomson effect. Adsorption capacity and kinetics for methylene blue varied significantly with pore structure, with materials possessing narrow mesopores showing superior dye uptake, and materials with larger mesopores and open-pore architecture exhibiting faster adsorption rates. This work demonstrates the tunability of mesoporous silica structure through precise control of synthesis conditions and highlights its potential in applications involving adsorption and phase phenomena in confined pore systems.

## 1. Introduction

Porous silica materials exhibiting mesoporosity have emerged as a class of highly functional materials owing to their large specific surface area, adjustable pore diameters, high thermal and hydrothermal stability, and tunable surface chemistry [1,2,3]. They show a well-organized mesoporous structure, uniform pore sizes, specific pore connectivities, wall thickness, and surface chemistry [3,4,5,6]. These features render them ideal candidates for diverse applications, including catalysis, separation processes, sensing, controlled drug delivery, and supports for functional nanomaterials and matrices for carbon nanomaterials [7,8,9,10,11,12].

Depending on the synthesis conditions and the nature of the structure-directing agent, a wide variety of mesostructures can be obtained (such as 2D hexagonal: MCM-41; SBA-15; 3D cubic: SBA-16; KIT-6; and worm-like or disordered mesopores) [13,14,15,16,17]. The synthesis of such materials typically involves a sol–gel process in the presence of structure-directing agents (SDAs), which act as templates guiding the assembly of the inorganic silica precursor into ordered or disordered porous architectures [18,19,20,21]. The earliest and most well-known examples of ordered mesoporous silicas, such as MCM-41, SBA-15, and FDU-type materials, were synthesized using surfactants and block copolymers [22,23,24,25,26]. These templating agents direct the self-assembly of micellar structures, which, upon condensation of silica around them and subsequent removal of the organic component (typically by calcination or extraction), leave behind mesostructured silica with well-defined pore arrangements.

Numerous studies have reported MCF-type materials synthesized using Pluronic P123 as the templating agent [27,28,29,30,31]. These materials exhibit a related porous architecture, though the pores are more regularly spherical and interconnected by circular openings or “windows”. The pore sizes in these systems typically range from 15 to 50 nm, with window diameters of 10–15 nm. Structurally, such materials resemble a three-dimensional network of randomly connected spherical voids.

The mechanism of formation of MCF-type silica materials is generally consistent with concepts related to the preparation of silica–organic composites for other structurally ordered silicas. The Liquid Crystal Templating theory [32] assumes the coexistence of two mechanisms: (i) structure is ordered before the addition of the silica source (after dissolving the copolymer in water, a liquid crystal structure is formed), and (ii) order is attained only after the addition of the silica source—starting with the formation of individual micelles, which gradually agglomerate, ultimately forming an ordered structure. According to the Folded Sheet model [33], a layered silica–copolymer structure is first formed (as a layered micelle) and then folds, forming three-dimensional channels. The cooperative formation mechanism [34] assumes the coexistence of differently shaped micelles and individual copolymer molecules. A shape reorganization through a change in this equilibrium state results from the addition of silica due to the formation of copolymer-silica precursor pairs linked by hydrogen bonds. Several theories attempt to explain the initial stage of the formation of mesoporous silica materials—the three selected theories above demonstrate the complexity of this process.

The nature of SDA plays a pivotal role in defining the final pore size, pore geometry, wall thickness, and degree of order in the mesoporous silica. For example, cetyltrimethylammonium bromide (CTAB), a cationic surfactant, typically directs the formation of hexagonally ordered mesopores with small diameters (~2–3 nm) characteristic of MCM-41 [35,36,37]. In contrast, non-ionic block copolymers, particularly the Pluronic-type triblock copolymers (EO–PO–EO), allow for the synthesis of mesoporous silica with larger pore sizes and tunable morphologies depending on the molecular weight and EO/PO ratio of the copolymer blocks [38,39,40,41]. Among Pluronic surfactants, the most widely studied are P123 (EO_20_–PO_70_–EO_20_) and F127 (EO_106_–PO_70_–EO_106_), which lead to SBA-15-type structures and three-dimensional cubic mesostructures, respectively [42,43,44]. However, recent studies have explored other members of the Pluronic family, such as Pluronic PE6800, PE9200, PE9400, and PE10500, which offer new opportunities for structural tuning due to their varied molecular weights and hydrophilic–lipophilic balance [45,46,47,48]. These lesser-known Pluronics represent a promising but underexplored set of templating agents for generating silica materials with unique structural features. They enable fine-tuning of pore size, connectivity, and wall thickness, which are critical parameters influencing the performance of mesoporous silica in practical applications. Moreover, the use of these SDAs under acidic or neutral synthesis conditions offers environmentally friendly and scalable pathways for material fabrication. Despite their potential, systematic comparisons of the effect of PE-series Pluronics on the properties of the resulting silica materials remain limited. Understanding how their molecular architecture translates to differences in mesostructure, porosity, and stability is essential for the rational design of functional silica-based materials.

The present study addresses this gap by investigating the synthesis of porous silica phases using Pluronic PE6800, PE9200, PE9400, and PE10500 as structure-directing agents for mesocellular foams, with particular attention to processes occurring within confined pore spaces. The research was carried out in several stages. In the first stage, the textural characteristics of the silica materials were examined and correlated with synthesis conditions, highlighting the influence of expander addition, aging temperature, copolymer type, and silica source. The second stage focused on phase transformations within confined pore spaces, preceded by a detailed structural characterization aimed at elucidating the mechanism of MCF-type mesoporous material formation. Finally, the adsorption kinetics of a model dye molecule was investigated and theoretically described for the synthesized mesocellular systems, establishing a relationship between adsorption behavior and the porous structure of mesocellular foams.

Considering the widespread use of porous materials and the influence of porosity on their functional properties, the study of processes occurring in confined pore systems is of fundamental importance. In this work, the adsorption and phase transition phenomena were investigated for mesoporous silicas with different porosity characteristics (in particular, taking into account the different geometric nature of the pores, including cylindrical and bottle-shaped slit pores). A series of mesocellular foam-type silicas was synthesized using less commonly studied Pluronic PE copolymers (PE6800, PE9200, PE9400, and PE10500), going beyond the widely investigated P123- and F127-based systems. By correlating the EO/PO ratio and molecular architecture of these copolymers with pore geometry, wall thickness, and structural order, this work demonstrates new pathways to precisely tailor mesoporous silica frameworks. The study results provide original insights into how synthesis variables such as copolymer type, pore expander addition, aging temperature, and silica precursor influence the development of bottle-shaped pores and mesocellular structures. Furthermore, linking these structural features with phase transformations in confined spaces and adsorption kinetics establishes a direct structure–function relationship, advancing the understanding of phase phenomena, transport, and adsorption in hierarchical porous materials. This integrated approach expands the design toolbox for mesoporous silica and highlights the potential of high-molecular-weight Pluronics as versatile structure-directing agents for advanced adsorption and separation applications.

## 2. Results and Discussion

### 2.1. Textural Characteristics of Silicas and Their Relationship to Synthesis Conditions

The effect of the synthesis method on the textural properties of silica materials was investigated by analyzing shapes of nitrogen adsorption/desorption isotherms, values of textural parameters, and pore size distributions derived from adsorption data. For comparative analysis, the synthesized materials were grouped into series, with samples within each series differing by a single synthesis variable. A summary of the most relevant textural parameters for each silica material, along with the corresponding synthesis conditions, is presented in Table 1.

#### 2.1.1. Effect of the Expander Addition

**Figure 1:**
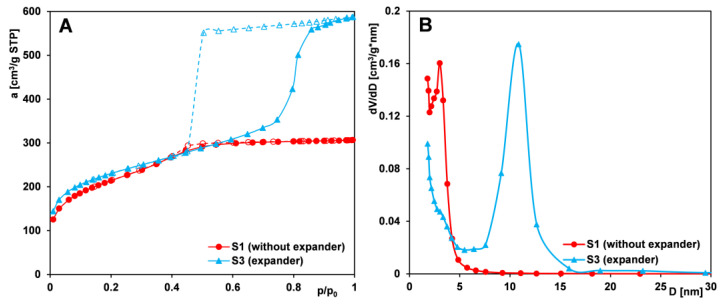
Comparison of (**A**) nitrogen adsorption/desorption isotherms and (**B**) pore size distributions derived from the adsorption branch of the isotherms for silica samples (S1 and S3) synthesized without and with an expander (TMB). Solid line and dash line correspond to adsorption and desorption data, respectively.

An expander is defined as a substance that is introduced into the reaction mixture during the synthesis of Pluronic-based silica materials, increasing the size of the forming micelles, thereby leading to an enlargement of the pore size in the resulting material. A common example of such an expander is 1,3,5-trimethylbenzene (TMB), a simple organic compound. The influence of expander addition on the textural properties of silica was examined using samples from Series I (S1 and S3). The corresponding nitrogen adsorption/desorption isotherms and pore size distributions for these samples are presented in Figure 1A and Figure 1B, respectively.

The nitrogen adsorption/desorption isotherms of both samples are similar up to p/p_0_ = 0.4 but diverge at higher pressures. For sample S1 (synthesized without the pore-expanding agent), the isotherm exhibits only a slight step associated with capillary condensation and a limited increase beyond p/p_0_ = 0.6. The overall shape of the isotherm corresponds to type IV according to the IUPAC classification, indicative of mesoporous materials with small mesopores. This is further supported by a pore size distribution (PSD) peak at 2–3 nm. The hysteresis loop suggests pores with a predominantly cylindrical geometry.

In contrast, the isotherm of sample S3 (prepared with the addition of a pore-expanding agent) displays a pronounced hysteresis loop beginning around p/p_0_ = 0.4, with a substantial nitrogen uptake above p/p_0_ = 0.6. The isotherm also corresponds to type IV, but with a well-defined H2-type hysteresis loop—typical of mesoporous materials with ink-bottle-shaped pores. The nearly vertical adsorption and desorption branches in this pressure range indicate a narrow pore size distribution. This is confirmed by the PSD function, which shows a sharp peak centered at approximately 11 nm. These results suggest that the addition of the expander during synthesis led to the formation of bottle-shaped mesopores with narrow openings and wide interiors. Concurrently, the textural properties of the material were significantly affected (Table 1), including an increase in average pore diameter and total pore volume, and are in accordance with the literature data regarding PE9400 and PE10500 copolymers and TMB as an expander [49]. Moreover, considering other studies and the general characteristics of the porous structure (indicated by the specific surface area S_BET_), the materials obtained by our research group (with S_BET_ values ranging from 650 to 940 m^2^/g) exhibit higher surface areas than those reported in the literature [50,51,52]. These results point to the successful optimization of the synthesis parameters employed in our work.

#### 2.1.2. Effect of Aging Temperature

**Figure 2:**
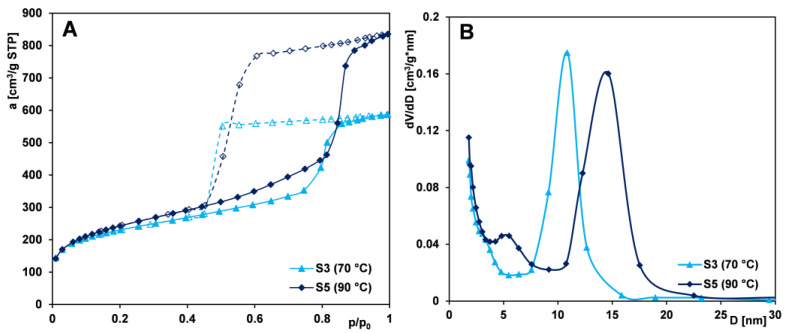
Comparison of (**A**) nitrogen adsorption/desorption isotherms and (**B**) pore size distributions determined from the isotherm adsorption branch for MCF samples (S3 and S5) whose precursors were aged at different temperatures. Solid line and dash line correspond to adsorption and desorption data, respectively.

**Figure 3:**
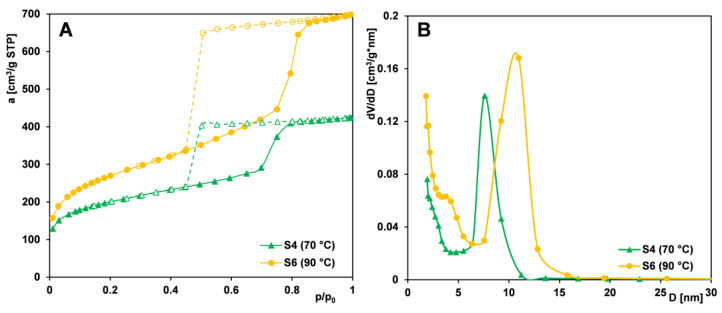
Comparison of (**A**) nitrogen adsorption/desorption isotherms and (**B**) pore size distributions determined from the isotherm adsorption branch for MCF samples (S4 and S6) whose precursors were aged at different temperatures. Solid line and dash line correspond to adsorption and desorption data, respectively.

Aging conditions of the precipitate play a key role in synthesizing mesostructured cellular foam (MCF) materials, as the silica framework forms via condensation and polymerization during this stage. Optimal aging typically involves acidic conditions and elevated temperatures. To assess the impact of aging temperature on textural properties, samples from Series IIA (S3, S5) and IIB (S4, S6) were compared. Figure 2A and Figure 3A present the nitrogen adsorption isotherms for samples whose precipitates were aged at different temperatures—70 °C and 90 °C. All samples exhibit a gradual increase in nitrogen uptake up to p/p_0_ = 0.7–0.8, followed by a pronounced adsorption step, indicating a narrow pore size distribution. Broad hysteresis loops suggest the presence of bottle-shaped pores in the silica’s structure.

For materials aged at elevated temperatures, an increase in nitrogen adsorption is observed at higher relative pressures, indicating a greater pore volume. This behavior can be attributed to partial dissolution of the silica framework and the reorganization of the polymer–TMB system (generation of additional mesopores). Broader hysteresis loops and PSD peaks reflect enlarged mesopores (Figure 2B and Figure 3B). PSD maxima shift with temperature—from 10.8 to 14.6 nm in Series IIA and from 7.6 to 10.8 nm in Series IIB. Additionally, samples aged at higher temperatures exhibit development of specific surface area (strong increase of S_BET_ for Series IIB, but moderate for Series IIA) and a strong increase in total pore volume compared to their counterparts aged at lower temperatures (Table 1).

Analysis of the desorption branches of the isotherms for Series IIA, prepared using the PE9400 copolymer, reveals that for sample S5, the desorption step occurs at higher relative pressures. This shift suggests that increasing the aging temperature leads to the widening of pore necks, likely due to partial dissolution of the silica framework in these regions. A different trend is observed for Series IIB, synthesized with the PE10500 copolymer. Here, minimal changes in hysteresis loop shape and size indicate a more mechanically stable structure with thicker pore walls. The narrower loops seen in Series IIB reflect a more rigid, alteration-resistant pore structure. The effect of changing the aging temperature for the PE10500 copolymer was studied previously in [53]. In those systems, increasing the aging temperature from 70 °C to 100 °C resulted in an increase in the specific surface area of about 5% and an increase in the total pore volume (V_t_) of about 57%. In the presented work, an increase in the specific surface area (S_BET_) of approximately 37% and a similar increase in V_t_ of about 63% were observed when the temperature was raised from 70 °C to 90 °C. These differences highlight the importance of the aging temperature at the stage of synthesis of the silica material. A more controlled or optimized aging process, as suggested in the presented work, can lead to significantly improved textural properties of the adsorbent, which could be beneficial for applications requiring high surface area and porosity of the materials. The precipitate aging temperature of 90 °C during the synthesis process was optimal for obtaining silica with the required features; a further increase in temperature does not result in positive effects on the development of the porous structure.

Overall, aging temperature significantly impacts the porous structure of Pluronic-templated silica. Higher temperatures (up to 90 °C) promote pore enlargement, enhance uniformity, and improve textural properties such as surface area, pore volume, and microporosity.

#### 2.1.3. Effect of Copolymer Type

In the synthesis of mesoporous silica foams (MCFs), Pluronic triblock copolymers serve as structure-directing agents. These copolymers consist of a central hydrophobic poly(propylene oxide) (PO) block flanked by two hydrophilic poly(ethylene oxide) (EO) segments. Variations in their molecular weight and EO/PO ratio influence micelle formation, solubilization behavior, and, consequently, the final porous architecture of the silica materials. To investigate the effect of copolymer structure on the resulting mesoporosity, samples from Series IIIA (S3, S4, S7, and S8) were synthesized using PE9400, PE10500, PE6800, and PE9200, respectively, with the addition of the pore-expanding agent TMB. Series IIIB (S1 and S2) included samples synthesized with PE9400 and PE10500, respectively, but without the addition of the TMB expander.

**Figure 4:**
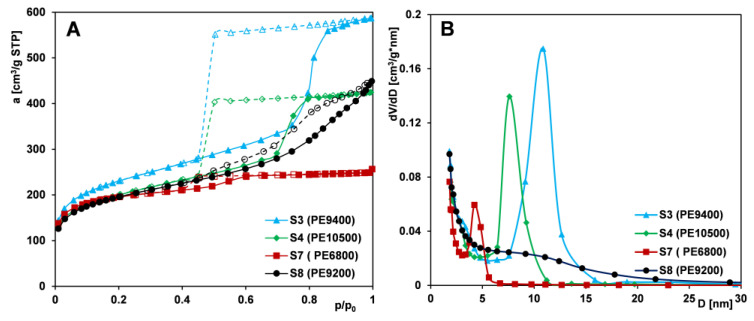
Comparison of (**A**) nitrogen adsorption/desorption isotherms and (**B**) pore size distributions determined from the isotherm adsorption branch for MCF-type samples (S3, S4, S7, and S8) synthesized using different polymers as pore-forming matrices. Solid line and dash line correspond to adsorption and desorption data, respectively.

Analysis of the adsorption isotherms for Series IIIA samples (Figure 4A) shows that sample S3 (synthesized with PE9400) exhibits the most developed porous structure, with the highest nitrogen uptake across all relative pressures. A sharp adsorption step and a symmetrical PSD peak at ~11 nm (Figure 4B) indicate a highly uniform mesoporous network.

Sample S4 (synthesized with PE10500) displays a similar isotherm but with a steeper adsorption jump at p/p_0_ ≈ 0.7 and a narrower hysteresis loop, suggesting even greater pore uniformity. Its plateau starts at a lower pressure (p/p_0_ = 0.8 vs. 0.9 for S3), indicating smaller pores and lower total pore volume and surface area.

For the remaining samples, S7 and S8, isotherms are very similar in the pressure range up to the point p/p_0_ = 0.4, after which they diverge significantly. For sample S7 (synthesized with PE6800), a slight increase in adsorption is observed at higher pressures, unlike for S8 (synthesized with PE9200), where a broad adsorption range indicates structural disorder. This is supported by its flat PSD curve. However, of all four materials, sample S7 shows the lowest textural parameters and a narrow PSD peak shifted to smaller diameters. When analyzing the shape of the hysteresis loop on the isotherms of the tested samples, it should be noted that the type of copolymer used for the synthesis of mesoporous materials also affects the pore architecture: (i) PE9400 and PE10500: H2-type (bottle-shaped pores); (ii) PE6800: H1-type (cylindrical pores); and (iii) PE9200: H4-type (slit-like pores).

The development of mesoporous structure strongly depends on the EO/PO ratio in Pluronics and the presence of TMB. The most pronounced synergistic effect between the copolymer and TMB is observed in the silica synthesized with PE9400. This is likely due to the high content of the PO block (60%) in the macromolecule, as this segment primarily determines the size of the micelle core. A higher proportion of PO results in the formation of larger micelles, which subsequently leads to the generation of larger pores within the silica structure. Slightly less favorable properties are exhibited by silica synthesized from the PE10500 copolymer, which contains equal proportions of PO and EO blocks. In contrast, the silica derived from the PE6800 copolymer, which has a low PO content (20%), shows poor structural order and the least favorable textural parameters. An exception to the overall trend is observed with PE9200-based silica, which displays structural disorder. This may be due to the insufficient length of the EO blocks, which are unable to stabilize a well-organized mesophase. The influence of the type of copolymer used on the characteristics of synthesized silica materials has been investigated by various research groups [54,55,56]. However, only our study reports such a wide variety of materials—differing in pore architecture, i.e., with bottle-shaped pores (based on PE9400 and PE10500), cylindrical pores (based on PE6800), and slit-like pores (based on PE9200). The shape of the pores can significantly influence the physical processes occurring within their spaces, which is of great importance for advanced adsorption and separation applications.

**Figure 5:**
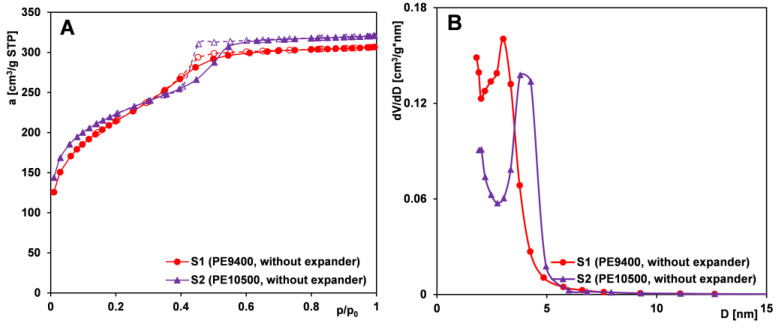
Comparison of (**A**) nitrogen adsorption/desorption isotherms and (**B**) pore size distributions determined from the isotherm adsorption branch for MCF-type samples (S1 and S2) synthesized using different polymers as pore-forming matrices. Solid line and dash line correspond to adsorption and desorption data, respectively.

Figure 5A,B shows nitrogen adsorption/desorption isotherms for silicas from Series IIIB (S1 and S2), both displaying an IUPAC type IV pattern with an H1-type hysteresis loop. At a relative pressure of 0.4, sample S2 shows a more distinct adsorption jump accompanying capillary condensation, indicating higher pore uniformity, which is confirmed by a narrower pore size distribution. The PSD curve for silica templated with PE10500 shifts toward larger diameters, suggesting wider pores compared to the PE9400-based material—opposite to the effect seen with TMB addition. This implies that, for materials obtained without a pore expander, pore size is determined by the molecular weight of the templating copolymer—higher weights yield larger pores and enhanced textural parameters (S_BET_, V_t_, and D_BJH ads_) but reduced microporosity.

Overall, mesoporous structure development depends heavily on the EO/PO ratio in the Pluronic copolymer and the presence of TMB. Among the tested variants, the silica material synthesized with the PE9400 copolymer in the presence of TMB exhibited favorable textural properties and the highest degree of structural order. In contrast, the PE9200 and PE6800 copolymers proved to be the least effective in forming well-defined MCF-type mesoporous silica.

#### 2.1.4. Effect of Silica Source

**Figure 6:**
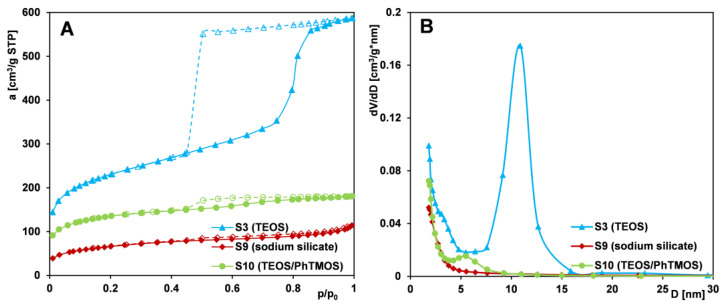
Comparison of (**A**) nitrogen adsorption/desorption isotherms and (**B**) pore size distributions determined from the isotherm adsorption branch for MCF-type samples (S3, S9, and S10) synthesized using different types of silica sources. Solid line and dash line correspond to adsorption and desorption data, respectively.

Another key factor influencing the porous structure of MCF materials is the type of silica source used. Figure 6A,B presents nitrogen adsorption–desorption isotherms and pore size distributions for Series IV samples (S3, S9, and S10) synthesized using tetraethyl orthosilicate (TEOS), sodium silicate, and a TEOS/trimethoxyphenylsilane (PhTMOS) mixture, respectively.

The obtained data indicate that sample S3 (synthesized with TEOS) exhibits the most developed and uniform porous structure, as reflected by the sharp adsorption step over a narrow relative pressure range and the narrow, symmetrical pore size distribution peak centered at approximately 11 nm. In contrast, sample S9 (synthesized with sodium silicate) displays a gradual increase in adsorption across the entire relative pressure range, with a more pronounced rise above p/p_0_ = 0.8. This behavior suggests low porosity and a relatively large external surface area. The hysteresis loop is poorly defined, and the corresponding pore size distribution is broad, indicating a wide range of small mesopores. Sample S10 (synthesized with TEOS/PhTMOS mixture) exhibits an isotherm similar to that of S9, although with higher adsorption at low relative pressures. A distinct hysteresis loop near p/p_0_ = 0.4 and a more defined adsorption step point to a modest improvement in pore uniformity. A small plateau appears above p/p_0_ = 0.9, and the pore distribution reveals a small, broad peak around 5 nm, indicating limited uniformity.

In summary, the use of sodium silicate or a TEOS/PhTMOS mixture as silica precursors leads to a marked decline in structural order and textural quality compared to TEOS-based materials. Variations in specific surface area, total pore volume, and average pore size highlight the critical influence of the silica source on MCF materials. Many studies indicate growing interest in replacing TEOS with cheaper and more environmentally friendly silicon sources [57,58]. This approach is important for industrial-scale production of materials, but it requires adjustments to the synthesis parameters, which are also evident in the materials we obtained using substitute silica precursors.

### 2.2. Mechanism of Formation of MCF-Type Mesoporous Materials

The synthesis of mesocellular foam (MCF) materials presented in this study relies on the use of amphiphilic Pluronic PE-type triblock copolymers, composed of a central hydrophobic poly(propylene oxide) (PO) segment flanked by two hydrophilic poly(ethylene oxide) (EO) chains. Under suitable pH conditions, the copolymers dissolve in water and self-assemble into micelles, with hydrophobic PO segments forming the core and hydrophilic EO chains extending outward. This amphiphilic behavior is crucial for templating the mesoporous structure of silica materials. The addition of 1,3,5-trimethylbenzene (TMB) expands micelles and promotes the formation of elongated, swollen structures, creating an oil-in-water microemulsion with copolymer/TMB droplets in an aqueous medium. When a silica precursor is added, silicate species deposit onto the micelles, hydrolyze, and condense to form a silica shell around the droplets. These silicate species interact via hydrogen bonding with the hydrophilic EO chains of the copolymer, initiating the formation of an organic–inorganic composite. During aging at high temperatures, the silica network condenses further, creating a rigid framework. After removing organic components via calcination, the final mesoporous silica foam is obtained.

The textural characteristics of the silica materials are strongly influenced by both the composition of the reaction mixture (swelling agent, type of copolymer, and silica source) and the synthesis conditions (aging temperature of the precipitate).

Silicas synthesized without the pore-expanding agent exhibit small mesopores with predominantly cylindrical geometry. The addition of the expander leads to the formation of a uniform structure with bottle-shaped mesopores, characterized by narrow openings and wide interiors. This is accompanied by an increase in both average pore diameter and total pore volume. Enhanced structural uniformity and further increase in textural parameters are observed when the aging temperature of the precipitate is raised; however, the pore shape remains unchanged (i.e., ink-bottle-shaped). This behavior is attributed to partial dissolution of the silica framework and reorganization of the polymer–TMB system. In the case of the PE9400-based silica, widening of the pore necks is observed with increasing temperature. This effect is not seen in the PE10500-based silica, indicating that the latter possesses thicker pore walls in this region and, consequently, a more thermally stable structure.

In the absence of a pore expander, the structure and size of the copolymer were the dominant factors controlling the final pore architecture. A higher molecular weight copolymer with longer hydrophobic and hydrophilic segments forms larger micelles in the reaction solution. As a result, the PE10500 copolymer generates larger pores in the silica framework than PE9400. Additionally, greater chain length tends to enhance micelle packing and promote pore connectivity in the final product, thereby improving textural parameters such as pore volume and surface area.

The development of the mesoporous structure in silica is strongly influenced by the copolymer structure and its interaction with TMB. The greatest pore uniformity and most favorable textural parameters are observed in silica synthesized using the PE9400 copolymer. This copolymer has a high content of the PO block (60%), which plays a key role in determining the micelle core size and, consequently, the pore size within the silica framework. Silica synthesized with the PE10500 copolymer, which contains equal proportions of PO and EO blocks, exhibits slightly worse textural parameters. In contrast, the PE6800 copolymer, containing only 20% PO, leads to a poorly ordered silica structure. An exception to this trend is the PE9200 copolymer (80% PO); using it leads to a lack of structural order in the resulting silica. This may be due to the short EO blocks being insufficient to stabilize a well-organized mesophase.

Using TEOS as a silica source results in a well-developed, uniform porous structure in MCF materials. Its high purity and well-defined molecular structure allow controlled hydrolysis and condensation during synthesis, forming a homogeneous silica network and promoting consistent ink-bottle-shaped pores. Partial replacement with PhTMOS introduces bulky phenyl groups, which disrupt silica network formation. TEOS/PhTMOS–derived material exhibits a lower degree of mesostructural organization and worse textural quality; the pore shape alternates from ink-bottle to cylindrical. Using sodium silicate, a more reactive source [59], leads to a loss of control over the templating process. It hydrolyzes and condenses rapidly, resulting in an irregular gelation process. Sodium silicate–derived material is characterized by poor porosity and a non-uniform pore structure.

Figure S2 shows a schematic representation of the MCF silica formation process.

### 2.3. Structural and Morphological Characteristics

**Figure 7:**
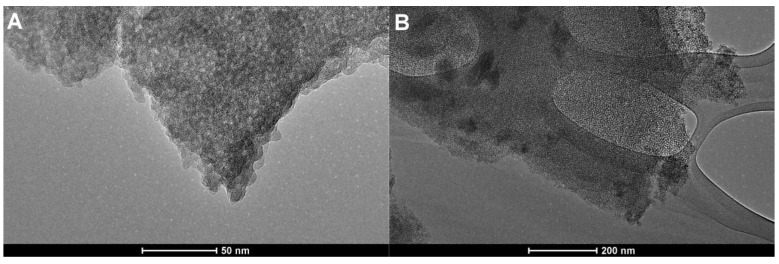

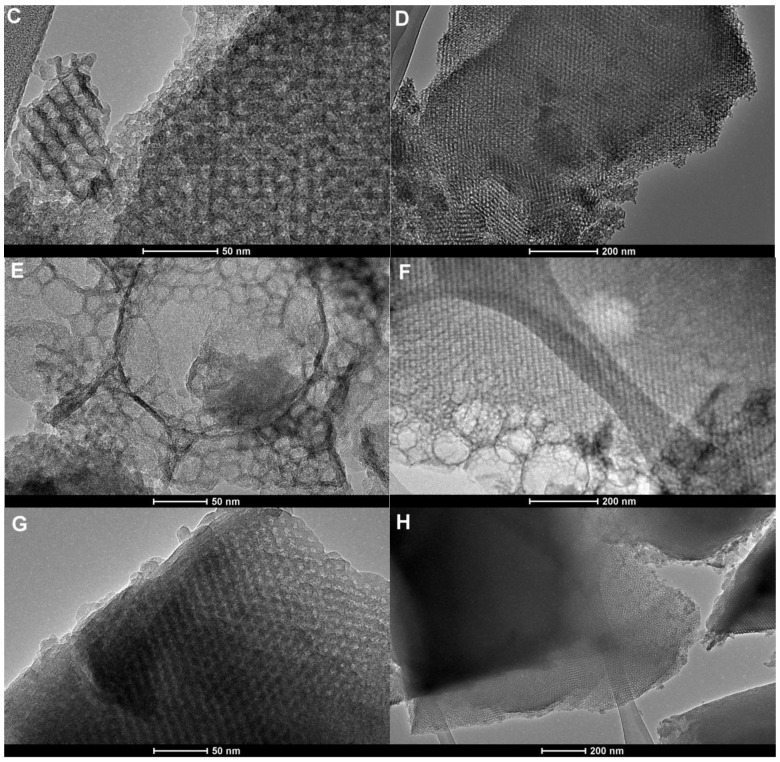
Transmission electron micrographs (TEM) of the MCF-type mesoporous materials under two magnifications: (**A**,**B**) (sample S1), (**C**,**D**) (sample S3), (**E**,**F**) (sample S5), and (**G**,**H**) (sample S7).

**Figure 8:**
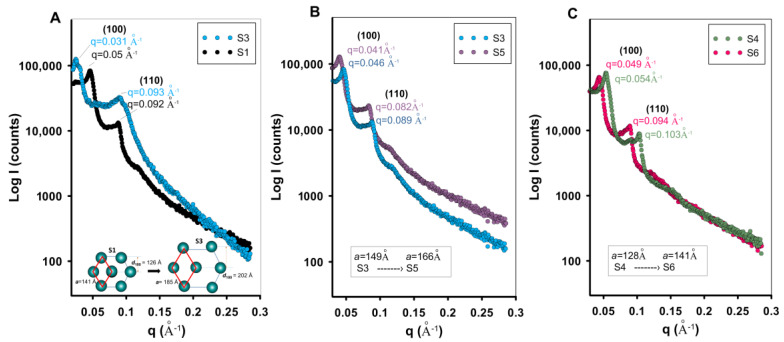
The SAXS curves of the investigated materials: (**A**) SAXS curves of the S1 and S3 samples and a 2D representation for a hexagonally ordered structure, (**B**) SAXS curves of the S3 and S5 samples, and (**C**) SAXS curves of the S4 and S6 samples. The interplanar distances for the (100) plane (d_100_) and lattice constants calculated as the average value for the (100) and (110) planes were placed on the figures.

Figure 7 presents a transmission electron microscopy (TEM) image of the synthesized silica materials. Figure 7A–D reveals TEM images of a sample from Series I (S1 and S3). In both cases, TEM images reveal well-developed porous architecture characteristic of mesocellular foams (MCFs), with a visible network of uniformly distributed pores. The contrast variations in the silica particle suggest a three-dimensional arrangement of pore cavities interconnected through narrower pore necks, confirming the “bottleneck” morphology typical of MCF-type materials for the S1 sample (without TMB expander) and greater “ink-bottle” morphology for the S3 sample (with TMB). For the S1 sample (Figure 7A and Figure 8B), the size of the mesopores appears to be relatively uniform across the particle, with estimated diameters in the range of approximately 3–5 nm, consistent with the dimensions observed in nitrogen sorption analyses (pore size distribution with peaks centered at 2 and 3 nm). Sample S3 (Figure 7C,D) reveals a network of regularly arranged mesopores and pores of greater size. This domain exhibits a relatively homogeneous distribution of spherical pores with diameters consistent with the mesopore range (~10 nm), confirming the material’s uniform porous morphology. In this case, spherical mesocages are connected by narrower pore openings, facilitating accessibility while maintaining a high surface area (792 m^2^/g). The bright regions correspond to the void spaces, and the darker contrast areas indicate that the silica framework is more clearly visible than in the S1 sample. The ordered contrast pattern visible in the left part of TEM image 8C suggests the presence of locally ordered mesocages, whereas the right part of the same image reveals a denser silica domain with less distinct pore orientation, indicating partial disorder or overlap of particles.

The TEM image of the S5 sample clearly shows the structural changes due to temperature increase (from 70 to 90 °C) and the presence of large, spherical mesocages arranged in a three-dimensional network. The image highlights the presence of voids and thin walls between adjacent mesocages, which promote efficient diffusion of guest molecules—a crucial factor in adsorption. The nearly isotropic pore morphology lacks significant long-range order. Compared to sample S3, this material shows larger and more spherical mesopores, with more distinct boundaries between individual mesocages. The TEM image for sample S5 should be directly correlated with the adsorption data. The nitrogen adsorption/desorption isotherm presented in Figure 2 exhibits the largest hysteresis loop, and the BJH pore size distribution defines pore dimensions at ~15 nm. Therefore, these data correlate well with the adsorption/desorption method and TEM electron microscopy.

Finally, the application of a copolymer with a higher proportion of hydrophilic elements (EO blocks), such as PE6800 (sample S7), at a temperature of 70 °C again creates a compact porous structure with pores of smaller diameters (Figure 7G,H), which is immediately visible in the adsorption/desorption isotherm, with a weakly defined hysteresis and a pore size distribution curve with a maximum below 5 nm (Figure 4).

The studied materials are structurally amorphous (except sample S9), without the presence of sharp peaks characteristic of crystalline structures (Figure S1A).

The obtained SAXS results (Figure 8) suggest that the use of 1,3,5-trimethylbenzene (TMB) as a swelling agent in the synthesis of MCF materials significantly influences their mesostructure, which is clearly reflected in the SAXS patterns. The low-angle pattern shows a well-defined signal below q = 0.05 Å^−1^ (below 0.75° of 2θ) for the (100) plane and the second signal (110) at q~0.09 Å^−1^ (~1.26° of 2θ) of the 2D hexagonal space group (p6mm). TMB, being a hydrophobic organic molecule, preferentially partitions into the hydrophobic core of the micelles formed by the block copolymer template, leading to micelle swelling and the formation of larger mesopores during the silica condensation process. Here, the lattice constant parameter of the hexagonal system increases from a = 141 Å to a = 185 Å for the S1 and S3 samples, respectively. A similar structural effect is generated by increasing the process temperature from 70 to 90 °C (Figure 8B,C), where the samples exhibit large-scale porosity and a bottle-shaped pattern. An increase in the lattice constant is observed for the samples maintained at higher temperatures. This increase, determined based on the lattice constant value, was from 149 Å to 166 Å for samples S3 and S5 and from 128 Å to 141 Å for samples S4 and S6. It is important to note that the structural order (presence of SAXS signals) is preserved despite the presence of the expander and the temperature increase. In the case of the remaining silica samples, a loss of structural order was observed for samples S8 and S9 (Figure S1B).

### 2.4. Phase Transformations in Confined Pore Spaces of Silicas

**Figure 9:**
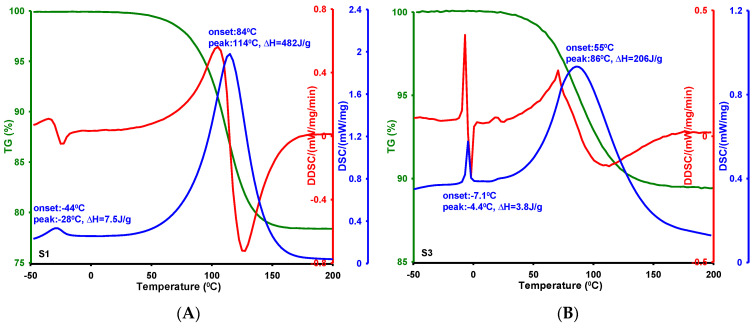

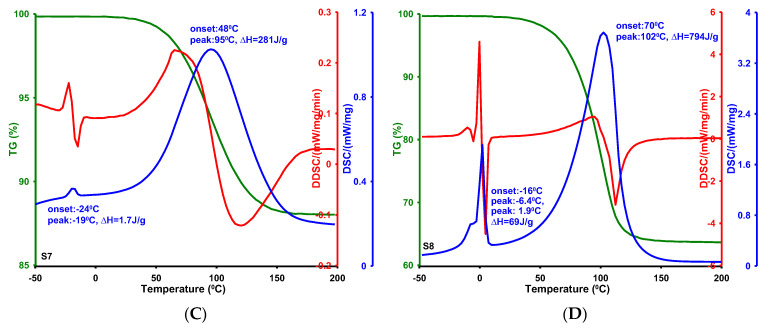
DSC curves for phase transformations occurring in closed pore spaces of silicas: S1 (**A**), S3 (**B**), S7 (**C**), and S8 (**D**).

Differentiation in the textural characteristics of MCF silicas is reflected in changes in the parameters and functions describing phase transitions occurring within the confined pores. This issue was analyzed based on differential scanning calorimetry (DSC) measurements, as each physical transformation is accompanied by heat absorption or release. Before measurements, the silica samples were subjected to a procedure of filling the pores with distilled water and removing it from the outer grain surface. Figure 9A–D presents thermograms obtained for samples S1, S3, S7, and S8. The DSC curve represents the heat flux exchanged between the tested sample and the environment as a function of temperature, while the DDSC curve (the first derivative of DSC) serves as an auxiliary function. The endothermic peaks in the DSC curves correspond, respectively, to the melting of frozen water bound in the pores and the evaporation of water, releasing it from the pores to the external environment. In the case of sample S8, the first peak is bimodal and may suggest both water within the pores and free water in the intergranular spaces.

Silica materials vary in their pore structure (size and shape), so each constitutes a unique thermodynamic system within which thermal transformations occur under specific temperature conditions. Analysis of the DSC curves allowed us to determine characteristic values of physical transformation temperatures for each sample: the onset temperature (T_on_, the intersection of the extrapolated baseline with the tangent to the rising part of the peak) and the phase transition temperature (T_max_, the temperature corresponding to the peak maximum). The change in phase transition enthalpy (ΔH), corresponding to the surface area between the DSC peak and the baseline, is also an important parameter. A detailed summary of characteristic temperatures and enthalpies specific to phase transformations occurring within the closed pores of silica materials is presented in Table 2.

**Figure 10:**
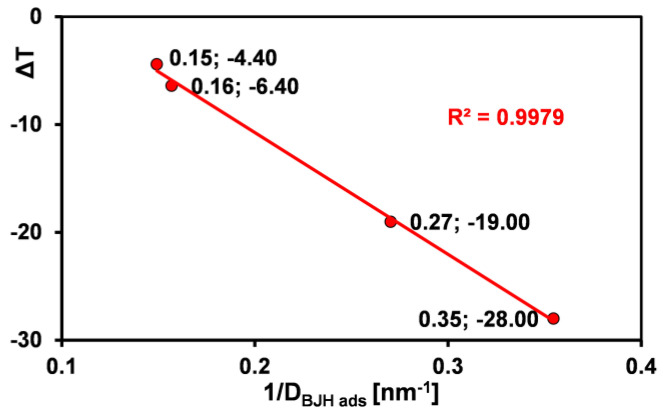
Linear dependence of depression in melting point (ΔT) on the average silica pore size.

For melting, the onset of the process is observed in the range of −44 to −7.1 °C, the phase transition point in the range of −28 to 4.4 °C, and the specific enthalpy values range from 1.7 to 69 J/g. When determining the enthalpy of fusion of ice for sample S8, the bimodal peak area was taken into account, meaning that this quantity has two components: (i) frozen water retained in the pores and (ii) frozen free water in the intergranular spaces. The maximum temperatures of the coexisting processes are −6.4 and 1.9 °C, respectively. The bimodal peak effect is not observed for the other samples due to the complete removal of water from the intergranular spaces during the material preparation stage. For this reason, the enthalpy of fusion for sample S8 differs significantly from the others. Considering the characteristic melting temperature values and the average pore size of the tested samples, certain correlations can be found. Generally, decreasing the pore size causes a decrease in both the melting onset temperature and the maximum phase transition temperature. This means that the depression in melting point (ΔT, ΔT = T_m_ − T_0_, where T_0_ and T_m_ are the melting temperature in the bulk liquid and the liquid in the pores) is inversely proportional to the pore size, as shown in Figure 10. However, the lack of correlation between specific enthalpy values and average pore size can be explained by the diversity of pore architecture of the tested samples (cylindrical, bottle-shaped, and slit-shaped pores), within which phase transitions are accompanied by different energetic effects.

The mechanism of lowering the melting point of ice in the tested materials can be linked to the unique properties of porous structures, meaning that water trapped in nanometric pores behaves differently from bulk water. The presence of silanol and siloxane groups on the internal surface of silica causes water molecules contained within the pore spaces to strongly adsorb via hydrogen bonds. If the pores are smaller, the adsorption layer is thinner, and the interactions of water with the pore surface are stronger. At the same time, lateral interactions between individual water molecules are weakened. Consequently, smaller pores further limit the ability to form a complete crystalline ice structure and thus significantly lower its melting point. Phenomenologically, the change in phase transition temperature is related to pore curvature via the Gibbs–Thomson equation. The results presented here are consistent with reports from scientific studies examining phase transitions (crystallization/melting) in the pore structures of hydrogels and silicas of the SBA-15 and MCM-41 types [60,61,62,63]. However, whereas earlier studies have investigated this correlation in silica materials with uniform pore architecture, the present work demonstrates that the relationship also holds for materials exhibiting distinctly different pore geometries, namely cylindrical, bottle-shaped, and slit-shaped pores.

In the case of the water-vapor phase transition, no correlation was found between the thermal effects and the silica pore size, confirming the complexity of the analyzed thermodynamic systems.

### 2.5. Adsorption of Methylene Blue on Selected Silicas

To evaluate the influence of the textural characteristics of silica materials on the processes at the solid–liquid interface, adsorption experiments with methylene blue were conducted. Due to its molecular size, this dye adsorbs to a limited extent on solids with a predominance of pores in the lower and mid-range of micropores; however, it is ideal for studying mesoporous structures. The physicochemical properties of the adsorbate are as follows: molecular weight—319.85 g/mol; pK_a_—2.6; 11.2; and water solubility—4%.

**Figure 11:**
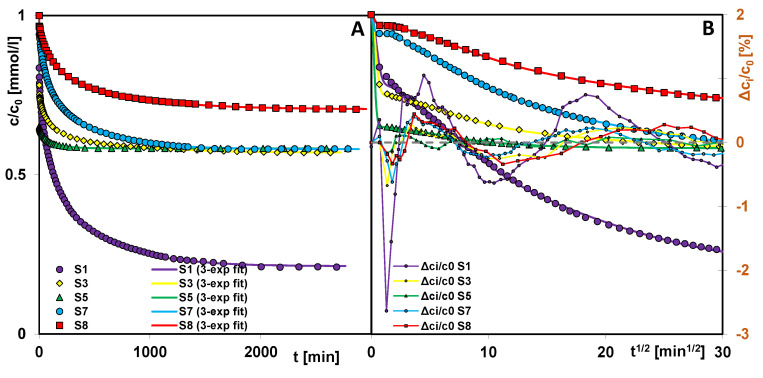

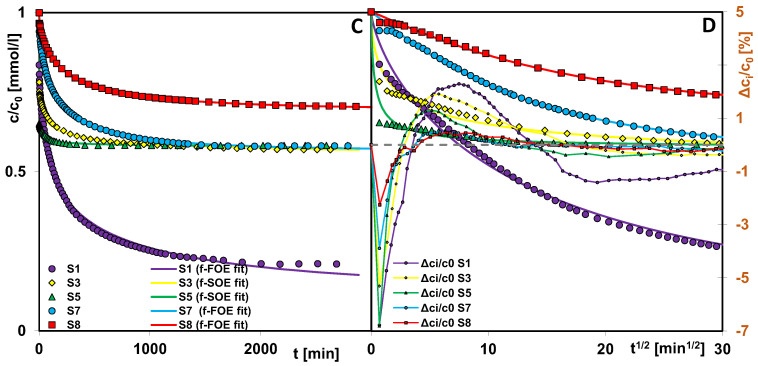
Comparison of the adsorption kinetics of methylene blue on mesoporous silicas in the coordinates: relative adsorbate concentration ~ time (**A**,**C**); relative adsorbate concentration~square root of time (**B**,**D**). The lines correspond to fits using the multiexponential equation (**A**,**B**) and the fractal MOE equation (**C**,**D**). The legend of the figures on the left is also valid for the figures on the right.

Studies of the adsorption process of methylene blue on synthesized silicas focused on its kinetics, as they allow for the determination of the rate and effectiveness of dye removal from solution by a given adsorbent. This is particularly important in the context of water purification in large-scale technological or environmental applications, as it allows for the selection of the appropriate adsorbent to shorten the purification time without compromising process efficiency. Figure 11A–D presents kinetic curves for methylene blue adsorption on selected silicas, as follows: relative adsorbate concentration versus time (A, C) and relative adsorbate concentration versus the square root of time (B, D).

**Figure 12:**
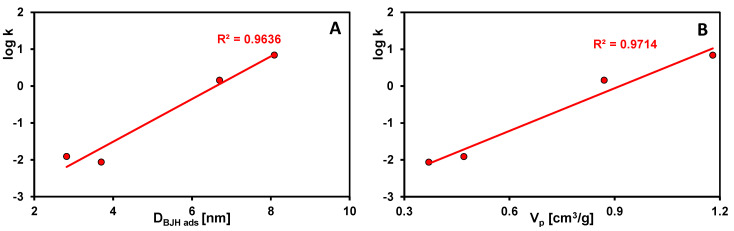
Linear dependences of the rate constant on the average pore size (**A**) and the rate constant on mesopore volume (**B**) in silica materials.

The rate of pollutant removal by adsorbents is closely correlated with their textural properties. The larger pore size (larger mesopore volume) of silica allows for a more time-efficient adsorption process. Based on the values of the rate constant (log k) and half-time (t_1/2_) determined in the results optimization procedure using the multi-exponential equation (Table 3), the individual adsorbents can be ranked according to their kinetic efficiency as follows: S5 > S3 > S1 > S7 > S8 (log k: 0.84, 0.16, −1.91, −2.06, −2.25; t_0.5_: 0.1, 0.5, 57, 80, and 123 min). Samples S5 and S3 are characterized by the presence of bottle-shaped pores with narrow entrances and wide interiors, while the application of a higher aging temperature to the first adsorbent precursor leads to widening of both the entrances and interiors of its pores. These changes enable very rapid diffusion of the adsorbate within the internal structure of the silica and occupation of adsorption-active sites. Material S1 obtained without the use of an expander has pores with a near-cylindrical shape and dimensions primarily in the lower mesopore range, which significantly slows the adsorption process. Even worse kinetic results obtained for sample S7 can be explained by the high proportion of micropores in the total porosity and, for sample S8, by the lack of structural order and the shape of pores formed in narrow gaps between the planes, which hinders the movement of the adsorbate. The linear relationships log k vs. D_BJH ads_ and log k vs. V_p_ for the four samples with the best fits are presented in Figure 12A and Figure 12B, respectively.

Adsorption of the dye on silica is possible primarily due to their different natures, respectively basic and acidic, and therefore relies on electrostatic interactions and hydrogen bonding. However, the mechanism of the dye adsorption process on porous bodies is more complex due to the specific behavior of its molecules in solution and at the solution–solid interface. This involves the possibility of coexistence in equilibrium of monomers, dimers, or larger molecules formed by the dye. Adsorption experiments for all tested systems were conducted until a near-equilibrium state was reached. Comparing the kinetics profiles presented in Figure 12, one can also determine the adsorption effectiveness of a given material showing specific textural properties. The highest methylene blue adsorption value was obtained for sample S1 (a = 0.037 mmol/g), significantly lower for samples S3, S5, and S7 (a = 0.020 mmol/g), and the lowest for sample S8 (a = 0.014 mmol/g). Taking into account the pore diameters, one can find a simple correlation between this factor and adsorption efficiency—the lower D the higher adsorption capacity (S1, S3, S5), which can be explained by stronger adsorption forces in the case of the S1 material characterized by cylindrical mesopores of small dimensions (greater dye molecule in relation to pore size favors adsorption). In the case of other samples, the additional factors should be also taken into account: differentiated pore shape, micropore share, values of pore volume, and specific surface area.

Better adsorption capacity of material S1 in comparison to the others may result from several interrelated factors, as follows: (i) the largest share of mesopores with diameters in the lower range, i.e., 2–3 nm (mesopores with the highest adsorption potential due to the overlap of adsorption forces of adjacent walls, which enables better packing of adsorbate molecules in the internal space of the pores or the formation of dimers and larger particles); (ii) the most favorable adsorption ratio of pore size to the size of the adsorbate molecule (methylene blue, as a relatively small molecule with a linear structure, is better adsorbed in smaller pores (small mesopores) than in larger pores (medium and large mesopores)); (iii) the smallest share of micropores (the lowest probability of the sieve effect preventing the diffusion of the dye into the pore space of the adsorbent with sizes smaller than the adsorbate). Points (i and ii) explain why silica S1 was a better adsorbent than materials S3 and S5, despite having a smaller specific surface area and total pore volume. These parameters for S1 are 754 m^2^/g and 0.47 cm^3^/g, respectively, while for S3 and S5, they are in the range of 792–852 m^2^/g and 0.91–1.29 cm^3^/g. It follows from the above that, in the case of methylene blue adsorption on porous bodies characterized by the presence of larger mesopores, part of the pore space remains unfilled. However, the size of many dye molecules exceeds the lower range of mesopores, so silicas S3 and S5 will be better suited for removing them from the solution.

Many models and kinetic equations were used to optimize the kinetic data, but the best fit was obtained for the multiexponential equation (m-exp) and the fractal MOE equation (f-MOE). Kinetic parameters determined using both equations and the quantities used to assess the accuracy of the adsorption kinetics description are summarized in Table 3. The multiexponential equation describes adsorption in a so-called compartment model with a series of first-order processes occurring in parallel and/or sequentially.

The fractal MOE equation was derived from the MOE equation by introducing an additional parameter (p-fractal coefficient) related to the non-ideality of the adsorption system. The greater the deviation of p from 1, the greater the influence of system fractality on the kinetics. The values of this parameter obtained fall within the range of 0.30–0.54, indicating a significant fractality effect. In the optimization procedure using the f-MOE equation, the f_2_ parameter was set to 0 or 1, so the equation for which f_2_ ∈ <0.1> is simplified to f-FOE or f-SOE, respectively.

The fit lines based on the multiexponential equation (m-exp) and the fractal MOE equation (f-MOE), along with the analysis of the percentage deviation between the fitted and experimental values (Δc_i_/c_0_= c_j,teor_ − c_j,exp_/c_0_, where c_j,teor_ is the theoretically calculated concentration at point “j”, c_j,exp_ is the experimentally determined concentration at the same point) are presented in Figure 11A,B and Figure 11C,D, respectively. Both optimization methods exhibit the greatest deviations in the initial stage of the experiment, where the adsorption process dynamics are most pronounced. However, the m-exp equation appears to be more flexible and provides a better fit across the entire experimental time range. Its higher accuracy is further supported by lower values of the determination coefficient 1 − R^2^ and the relative standard deviation SD(c)/c_0_. Nevertheless, the values of the most important kinetic parameters, such as the logarithm of the rate constant (log k), the half-time (t_0.5_), and the total adsorbate uptake (u_eq_) obtained from both methods are comparable.

### 2.6. Visualization of Dye Adsorption on Silica Grains

Fourier Transform Infrared (FTIR) mapping, as a powerful analytical technique that combines infrared spectroscopy with spatially resolved imaging, was utilized to provide detailed chemical information across the surface of a sample. By collecting a full infrared spectrum at each point within a defined area, FTIR mapping enables visualization of the spatial distribution of functional groups and molecular components such as dye molecules on the adsorbent surface. In the case of mesocellular foam (MCF) silica materials, this technique allows for the direct visualization of dye adsorption at the microstructural level, providing insight into how and where molecules are incorporated within the intergranular network.

**Figure 13:**
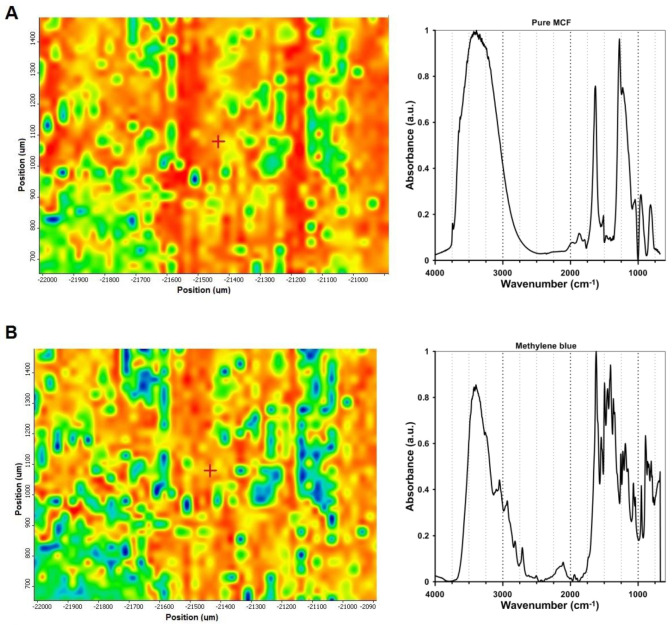

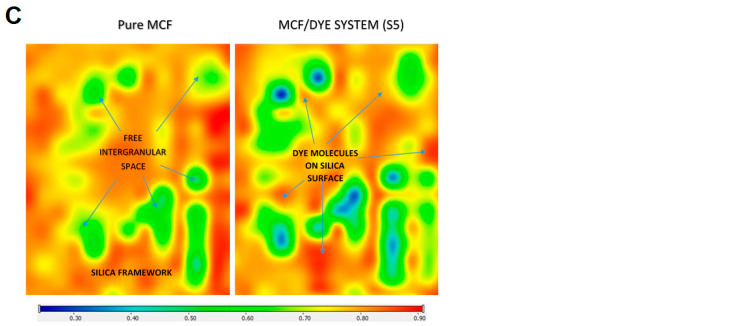
Correlation map of the components on S5/methylene blue system by FTIR mapping: (**A**) microscopic image of the mapped area 1100 µm × 800 µm as a distribution correlation map of silica spectra (generated from the point marked with a red cross) on S5/methylene blue system; (**B**) microscopic image of the mapped area as distribution correlation map of dye spectra (in the S5/methylene blue system (generated from the point marked with a red cross); (**C**) FTIR mapping of MCF/dye system (S5 sample). The left image presents the correlation map relative to the silica reference, and the right image presents the correlation map relative to the methylene blue adsorbate as the reference.

Figure 13A illustrates the correlation map of the S5/dye sample in relation to the pure MCF phase. The chemical maps were generated based on the intensity of selected vibrational bands characteristic of the dye (1260 cm^−1^) and silica framework (1200 cm^−1^), providing spatial insight into the distribution of molecular species across the sample surface. Here, FTIR spectra collected across the sample area predominantly display characteristic vibrational bands of the silica framework (Si–O–Si asymmetric and symmetric stretching modes, typically at ~1100 cm^−1^), confirming the presence of the intergranular space (blue and green areas). Figure 13B represents the correlation maps of the S5/dye sample in relation to methylene blue in the MCF matrix. In this case, FTIR microscopy enables detection of new absorption bands corresponding to functional groups of the dye (e.g., aromatic C=C stretching, N–H bending, or C–N stretching modes in the 1500–1600 cm^−^^1^ region). By performing chemical surface mapping based on these marker bands, it becomes possible to visualize the spatial localization of the dye molecules within the silica framework around the still empty intergranular space.

Figure 13C presents a comparative FTIR mapping analysis of mesocellular foam (MCF) silica before and after the adsorption of dye molecules from one selected area of the S5/methylene blue sample. The dye-loaded MCF sample (right panel) exhibits a pronounced and spatially resolved increase in IR signal intensity, particularly in the regions corresponding to the characteristic vibrational modes of the adsorbed dye molecules (e.g., aromatic ring vibrations, C–N or N–H stretching). The mapping reveals localized areas of strong signal intensity, indicating successful adsorption of the dye into specific domains of the silica framework (according to silica grains). This suggests that the dye molecules are not only present but are preferentially concentrated in silica framework regions, possibly due to variations in surface chemistry.

The presented data from FTIR microscopy with chemical mapping provide direct evidence of dye incorporation on the silica surface, enabling correlation between the material’s porous and grain architecture and adsorption behavior.

## 3. Materials and Methods

### 3.1. Materials and Chemicals

The non-ionic triblock copolymers of the Pluronic series were obtained from BAFS (West Port Arthur Road, Beaumont, TX, USA). Hydrochloric acid (35–38% purity) was supplied by Polish Chemical Reagents (POCh, Poznań, Poland). The 1,2,4-trimethylbenzene (TMB, 98% purity), tetraethyl orthosilicate (TEOS, ≥99.0%), trimethoxyphenylsilane (PhTMOS, ≥97.0%), and sodium silicate solution (extra pure) were purchased from Sigma-Aldrich (Poznań, Poland). For the synthesis of mesocellular foam (MCF) materials, four Pluronic-type copolymers (EO)x(PO)y(EO)x—PE9200, PE9400, PE10500, and PE6800—were selected (West Port Arthur Road, Beaumont, TX, USA). Physicochemical properties of the selected copolymers are summarized in Table S1.

### 3.2. Synthesis of MCF Materials

Mesocellular foams (MCFs) were synthesized using a procedure adapted from previously reported methods [64,65]. The syntheses were conducted under strongly acidic conditions, employing nonionic triblock copolymers of the Pluronic type and one of the following silica sources: tetraethyl orthosilicate (TEOS), a mixture of TEOS and trimethoxyphenylsilane (PhTMOS), or sodium silicate (water glass). In a typical synthesis, 10 g of a selected copolymer (PE9200, PE9400, PE10500, or PE6800) was dissolved in 136 g of 1.6 M hydrochloric acid. For selected formulations, 10 g of 1,3,5-trimethylbenzene (TMB) was added as a pore-expanding agent. The solution was gently stirred at 35 °C for 45 min. Subsequently, 34 g of the silica source, either pure TEOS, a TEOS/PhTMOS mixture (82/18 wt%), or sodium silicate, was introduced into the reaction mixture. Stirring continued for an additional 20 h. The resulting precipitate was aged in the mother liquor for 24 h at either 70 °C or 90 °C, depending on the desired material properties. The final product was recovered by filtration, thoroughly washed with distilled water until a neutral pH was achieved, and dried under ambient conditions. The dried material was then calcined at 500 °C for 6 h to remove the organic template.

### 3.3. Experimental Techniques

#### 3.3.1. Material Characterization

Nitrogen adsorption/desorption isotherms at 77 K were measured on an ASAP 2405 (Accelerated Surface Area and Porosimetry) apparatus from Micromeritics Instrument Co. (Norcross, GA, USA). Immediately before measurement, the test samples were degassed (10^−4^ mmHg) at 473 K. The adsorption data were used to determine the BET specific surface area, S_BET_, and total pore volume, V_t_, using standard methods (S_BET_ was determined from the linear BET relationship, while V_t_ was determined from the adsorption value at a relative pressure p/p_0_ = 0.98). The external surface area, S_ext_, and mesopore volume, V_p_, were determined by the αs method using LiChrospher Si-1000 adsorbent (Sigma-Aldrich, Poznań, Poland) as a standard [66]. Pore volume distributions were determined using the Barrett–Joyner–Halenda (BJH) method [67].

X-ray diffraction measurements of the powder materials investigated in this study were carried out using a PANalytical Empyrean powder diffractometer (currently Malvern PANalytical, Almelo, The Netherlands), equipped with a reflection geometry setup and a high-performance X-ray source. The measurements were performed using a copper (Cu) anode tube, which emits X-ray radiation with a wavelength of λ = 1.5418 Å. The diffractometer was operated at 40 kV and 40 mA. Data were collected in reflection mode within a 2θ scanning range of 10° to 80°, with a step size of 0.02° and a counting time of 1 s per step. A PIXcel3D multi-channel detector (Malvern PANalytical, Almelo, The Netherlands) was used to collect the diffraction patterns. Powder samples were evenly spread onto the sample holder and gently pressed to obtain a flat and homogeneous surface. All measurements were conducted at room temperature. This method enabled the identification of crystalline phases and the evaluation of structural ordering in the synthesized silica-based materials.

Small-angle X-ray scattering (SAXS) measurements were performed using a Malvern PANalytical Empyrean diffractometer (PANalytical, Almelo, The Netherlands), configured to measure scattering in the low-angle range characteristic of nanostructured materials. The experiment was conducted using a copper (Cu) anode X-ray tube with a wavelength of λ = 1.5406 Å, operated at 45 kV and 40 mA. The SAXS configuration employed a collimated transmission beam geometry, achieved through a system of slits and parabolic mirrors. The scattering data were collected using a position-sensitive PIXcel3D detector in the angular range of 0.1° to 5° 2θ, with a fine step size of 0.001°. Measurements were carried out in transmission mode, with samples prepared in the form of thin films. SAXS analysis allowed for the investigation of the mesostructural order and pore architecture, providing information on periodicity and structural domains in the range of 1–100 nm.

Transmission electron microscopy (TEM) analysis of selected silica materials was performed using a Tecnai G2 20 X-TWIN electron microscope (FEI Company, Tokyo, Japan), equipped with a LaB_6_; electron source. The instrument operated at an accelerating voltage of 200 kV. For sample preparation, the silica powders were dispersed in 99.8% ethanol to form a homogeneous slurry. The resulting suspension was drop-cast onto a 200-mesh copper grid coated with lacey formvar and stabilized with carbon. After drying, the prepared grids were introduced into the microscope.

Chemical mapping was carried out using a Nicolet iN10 MX FTIR microscope (Thermo Scientific, Waltham, MA, USA) equipped with a motorized XY stage and a liquid-nitrogen-cooled MCT (Mercury Cadmium Telluride) detector. The measurements were performed in reflection mode. The resulting chemical maps were generated and processed using Omnic Specta™ software (version 8.1), based on spectral correlation in relation to the primary spectrum of the silica carrier and the pure dye.

#### 3.3.2. Measurements of Liquid Phase Transitions

Measurements of phase transitions of liquids in solid pores were performed using the differential scanning calorimetry technique (DSC) using STA 449 Jupiter F1 apparatus (Netzsch, Selb, Germany). Before measurement, each silica sample was (i) vacuum dried at 100 °C for 24 h, (ii) immersed in distilled water in a 1:10 ratio, (iii) shaken in a New Brunswick incubator shaker at 25 °C for two days, (iv) filtered, and (v) dried at room temperature for 2 h. 10 mg of the prepared sample was weighed and encapsulated in an alumina crucible. Measurements were conducted in a helium atmosphere in the temperature range from -100 °C to 500 °C, at a heating rate of 10 °C/min.

#### 3.3.3. Dye Adsorption Kinetics Measurements

The kinetic measurements were conducted for methylene blue adsorbed on selected silicas. The experiment began at the moment of introducing 0.05 g of silica into a vessel containing 50 mL of dye solution with a concentration of 0.047 mmol/L. Using the special Cary instrument software, version 2 (Varian, Melbourne, Victoria, Australia), a sample of the solution was automatically collected at preset time intervals from the adsorption system and directed to a flow cell, where the spectrum was measured. After each measurement, the sample was returned to the adsorption system. Throughout the experiment, the adsorption system was maintained at 25 °C (Thermostat Ecoline RE 207, Lauda, Germany) and stirred at 110 rpm. Based on the measurements, kinetic profiles were generated and then optimized using the multi-exponential equation and the fractal-like MOE equation [68,69,70,71]. For specific parameter values, the fractal-like MOE equation takes the simplified form of fractal-like FOE or fractal-like SOE. General equations and half-time expressions used in kinetic models are presented in Table S2.

## 4. Conclusions

In this work, mesoporous silica materials with diverse structural properties were obtained by using different Pluronic copolymers, changing synthesis conditions, and varying the composition of the reaction mixture.

- All copolymers (PE6800, PE9200, PE9400, PE10500) allow the production of mesoporous materials with lower and medium mesopore sizes. The material synthesized using the PE9400 copolymer exhibits the most favorable structural parameters and high uniformity in pore size. The PE9200 and PE6800 copolymers were the least effective. The type of matrix plays a significant role in the formation of the porous structure, which is related to their properties and structure and the number of central poly(isopropylene oxide) (PO) chains and poly(ethylene oxide) (EO) side segments. The copolymers with a high content of the PO block enable optimal utilization of the pore-enlarging agent, TMB. In the absence of an expander, pore sizes are determined by the dimensions of the copolymer macromolecules.
- Raising the aging temperature improves the structural parameters of the tested materials. The sample obtained using the PE9400 copolymer and aged at 90 °C is characterized by the largest pore volume and average pore size (1.29 cm^3^/g and 8.09 nm, respectively), resulting from partial dissolution of the silica framework and generation of additional mesopores. The sample aged at 90 °C, but synthesized using the PE10500 copolymer, has a more rigid silica framework (thermally stable), so its porous structure is characterized by the greatest specific surface area (BET method—944 m^2^/g) of all the samples tested. Pore enlargement as a temperature effect is accompanied by the maintenance of uniformity in the size distribution.
- The addition of an expander (TMB) to the reaction mixture enables the production of materials with bottle-shaped mesopores (narrow openings and wide interiors), larger pore volumes, and larger pore sizes. Samples obtained without the use of TMB have pores with cylindrical geometry.
- Comparing silicas obtained from three different silica sources (TEOS, sodium silicate, TEOS/PhTMOS mixture), one can identify the sample obtained from TEOS as the one with the most favorable structural parameters.
- The wide-angle XRD patterns revealed amorphous features of the synthesized silicas, indicative of the lack of long-range order. The results demonstrated that the use of a swelling agent effectively increased the lattice constant, thereby enlarging the mesopore size. Similarly, raising the synthesis temperature led to an increase in the lattice constant, confirming that both TMB addition and thermal conditions play a crucial role in tailoring the pore architecture. Importantly, the mesostructural order was preserved across most samples despite these modifications. The study shows the effective modulation of mesostructural features in silica materials through synthesis parameters such as swelling agent content and temperature, while emphasizing the complementary nature of XRD, SAXS, and nitrogen sorption in evaluating porous materials.
- The textural properties of MCF-type silica materials significantly influenced the thermal behavior of water confined within their porous structures. Differential scanning calorimetry (DSC) revealed that melting and evaporation processes are sensitive to variations in pore size and shape, with the onset and maximum temperatures of melting showing a clear correlation with average pore diameter. Specifically, smaller pores result in lower melting points due to enhanced water–surface interactions and restricted crystallization, consistent with the Gibbs–Thomson effect. These findings underscore the importance of pore architecture in governing phase transition behaviors within confined systems.
- Pore size, shape, and structural order of silicas influenced the adsorption behavior of methylene blue. Kinetic studies showed that materials with larger mesopores and open-pore architecture exhibited faster adsorption rates due to improved molecular diffusion and easier access to adsorption sites. However, the highest adsorption was observed for silica S1, attributed to the optimal ratio of pore size to adsorbate dimensions and a significant presence of small mesopores, which enhance packing efficiency and molecular interactions. Despite its lower surface area and total pore volume, its structure allows for more effective retention of methylene blue molecules. These findings offer practical guidance for tailoring silica adsorbents for dye removal and other environmental purification applications, with specific materials better suited for targeting molecules of different sizes or diffusion characteristics.

## Figures and Tables

**Table 1:** Textural parameters for the synthesis of MCF silica materials.

Silica	S1	S2	S3	S4	S5	S6	S7	S8	S9	S10
Type of copolymer	PE9400	PE10500	PE9400	PE10500	PE9400	PE10500	PE6800	PE9200	PE9400	PE9400
Type of pore expander	-	-	TMB	TMB	TMB	TMB	TMB	TMB	TMB	TMB
Silica source	TEOS	TEOS	TEOS	TEOS	TEOS	TEOS	TEOS	TEOS	sodium silicate	TEOS/PhTMOS
T_aging_ ^1^ [°C]	70	70	70	70	90	90	70	70	70	70
S_BET_ ^2^ [m^2^/g]	754	759	792	689	852	944	651	669	232	457
S_ext_ ^3^ [m^2^/g]	4	4.5	19	14	55	25	9	80	27	4
V_t_ ^4^ [cm^3^/g]	0.47	0.50	0.91	0.66	1.29	1.08	0.40	0.69	0.17	0.28
V_p_ ^5^ [cm^3^/g]	0.47	0.49	0.87	0.63	1.18	1.03	0.37	0.54	0.12	0.27
V_p_/V_t_ ^6^	0.99	0.98	0.96	0.95	0.91	0.96	0.93	0.78	0.69	0.97
D_BJH ads_ ^7^ [nm]	2.82	3.25	6.70	5.39	8.09	6.01	3.70	6.37	4.28	3.56

^1^ Process aging temperature. ^2^ Specific surface area calculated using the BET method. ^3^ External surface area determined from α_s_ plot. ^4^ Total pore volume determined from adsorption at the relative pressure p/p_0_ = 0.98. ^5^ Primary mesopore volume determined from α_s_ plot. ^6^ Mesopore contribution. ^7^ Average pore size calculated from adsorption data using the BJH method.

**Table 2:** Results of studies on the thermal effects of water in closed pores of silica materials.

Silica	T_1 on_ ^1^ [°C]	T_1 max_ ^2^ [°C]	ΔH_1_ ^3^ [J/g]	T_2 on_ ^4^ [°C]	T_2 max_ ^5^ [°C]	ΔH_2_ ^6^ [J/g]	D_BJH ads_ ^7^ [nm]
S1	−44	−28	7.5	84	114	482	2.82
S3	−7.1	−4.4	3.8	55	86	206	6.70
S7	−24	−19	1.7	48	95	281	3.70
S8	−16	−6.4/1.9	69	70	102	794	6.37

^1,4^ Onset temperature for melting and evaporation processes, respectively. ^2,5^ Peak maximum for melting and evaporation processes, respectively. ^3,6^ Specific enthalpy for melting and evaporation processes, respectively. ^7^ Average pore size calculated from adsorption data using the BJH method.

**Table 3:** Comparison of kinetic parameters determined based on the multi-exponential equation (m-exp) and the fractal MOE equation (f-MOE).

Silica	Fit	f_2_/p	log k ^1^	t_0.5_ [min]	u_eq_	a [mmol/g]	SD(c/c_0_) [%]	1-R^2^
S1	3-exp	-	−1.91	57	0.79	0.037	0.61	7.89 × 10^−4^
	f-FOE	0/0.36	−2.31	74	0.89	-	1.78	7.30 × 10^−2^
S3	3-exp	-	0.16	0.50	0.43	0.020	0.19	4.38 × 10^−4^
	f-SOE	1/0.30	0.70	5	0.50	-	1.31	2.42 × 10^−2^
S5	3-exp	-	0.84	0.10	0.42	0.020	0.07	8.63 × 10^−5^
	f-SOE	1/0.39	1.00	0.10	0.43	-	1.40	4.01 × 10^−2^
S7	3-exp	-	−2.06	80	0.42	0.020	0.20	2.32 × 10^−4^
	f-FOE	0/0.52	−2.24	86	0.43	-	0.63	2.50 × 10^−3^
S8	3-exp	-	−2.25	123	0.30	0.014	0.19	3.67 × 10^−4^
	f-FOE	0/0.54	−2.43	135	0.30	-	0.40	1.74 × 10^−3^

^1^ k: k_1_-f-FOE; k_2_-f-SOE; k_avg_-m-exp (k_avg_ = ln2/t_0.5_), t_0.5_: t_0.5avg_-m-exp (t_0.5avg_—overall adsorption half-time determined numerically).

## Data Availability

The data presented in this study are available upon request from the corresponding author.

## Supplementary Materials

The following supporting information can be downloaded at: https://www.mdpi.com/article/10.3390/ijms26189255/s1.
