# Supplementary material for: Mesoporous Silicas of Well-Organized Structure: Synthesis, Characterization, and Investigation of Physical Processes Occurring in Confined Pore Spaces
Source: Int J Mol Sci. 2025 Sep 22;26(18):9255. doi: 10.3390/ijms26189255 (PMC12470678; doi:10.3390/ijms26189255)
Supplement: Supplementary file 1 [file ijms-26-09255-s001.zip › ijms-3818973-supplementary.pdf]

International Journal of
Molecular Sciences

MDPI

Mesoporous Silicas of Well-Organized Structure: Synthesis, Characterization, Investigation of Physical Processes Occurring in Confined Pore Spaces

Magdalena Blachnio[1,*], Malgorzata Zienkiewicz-Strzalka[1] and Anna Derylo-Marczewska [1]

[1] Department of Physical Chemistry, Institute of Chemical Sciences,Maria Curie-Sklodowska University, Maria Curie-Sklodowska Square 3, 20-031 Lublin, Poland; malgorzata.zienkiewicz-strzalka@mail.umcs.pl (M.Z.-S.); anna.derylo-marczewska@mail.umcs.pl (A.D.-M.)

* Correspondence: magdalena.blachnio@mail.umcs.pl; Tel.: +48-0815375637

SUPPLEMENTARY INFORMATION

All synthesized silicas were investigated by using the powder X-ray diffraction technique. The diffraction patterns are presented in Figure S1A. One can state that the studied materials are structurally amorphous (except sample S9), without the presence of sharp peaks characteristic of crystalline structures. Instead, broad, diffuse bands are observed, which is the result of the lack of long-range order in the amorphous structure. Amorphous silica exhibits a broad, low-intensity diffraction halo centered around 2θ = 15–30°. This feature arises from the short-range order between silicon and oxygen atoms in the silica network, reflecting the absence of long-range periodicity. The exact position and width of this amorphous signal may vary slightly depending on the synthesis conditions, porosity, and degree of condensation of the silica walls. In this case, for all material series, the amorphous signal is of similar intensity and position. The degree of amorphization for materials S1–S10 is generally comparable. The only notable exception is sample S9, synthesized using water glass as the silica source. In this case, distinct peaks observed at approximately 28.4° and 47.5° (2θ) correspond to the (111) and (220) reflections of the crystalline silicon phase (CPDS card No. 39-1346).

Small-Angle X-ray Scattering (SAXS) was employed to investigate the mesostructural ordering of MCF S1-S10 silica materials. MCFs typically exhibit a three-dimensional, sponge-like mesoporous network with large mesocages interconnected by narrower pore windows. In this case, SAXS provides critical information about the periodicity and the degree of ordering within the investigated mesostructures. In the case of the silica samples S7-S10 (Figure S1B), a loss of structural order was observed for samples S8 and S9. This observation aligns with the nitrogen adsorption/desorption analysis, which showed the absence of a distinct maximum in the pore size distribution curves and the presence of isotherms with narrow hysteresis loops (Figure 4 and Figure 6). In all cases, small-angle X-ray scattering (SAXS) results were consistent with the adsorption data. However, the lattice constants determined from SAXS were generally slightly larger than the pore sizes obtained from the distribution functions. This discrepancy arises because the lattice constant from SAXS corresponds to the center-to-center distance between adjacent pores, encompassing both the pore diameter and the silica wall thickness. In contrast, the BJH model reflects only the internal pore diameter, excluding the wall structure. Therefore, nitrogen adsorption measures the pore size, while SAXS provides information about the overall structural periodicity.

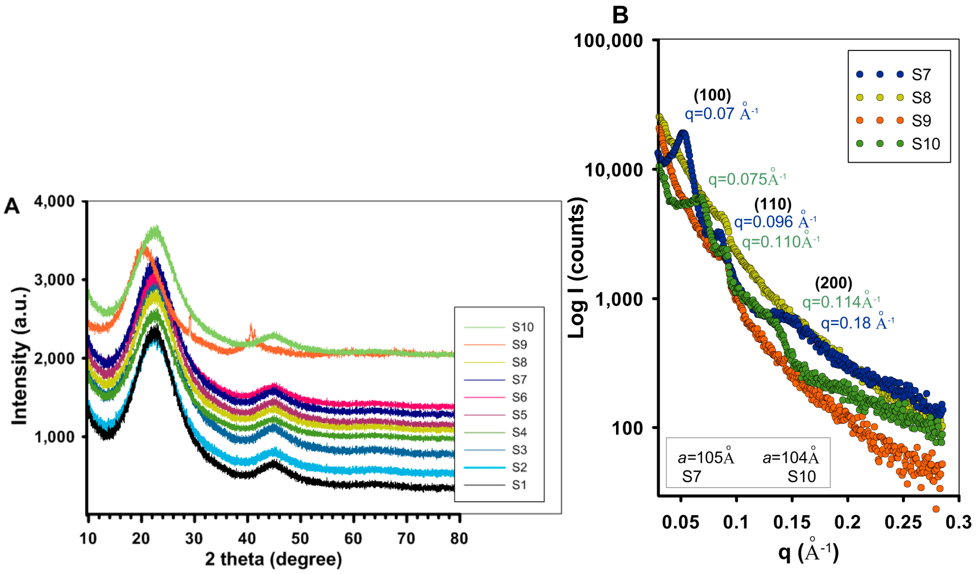

Figure S1. (A) Wide-angle XRD patterns of the MCF materials S1-S10, (B) The SAXS analysis for the S7-S10 samples.

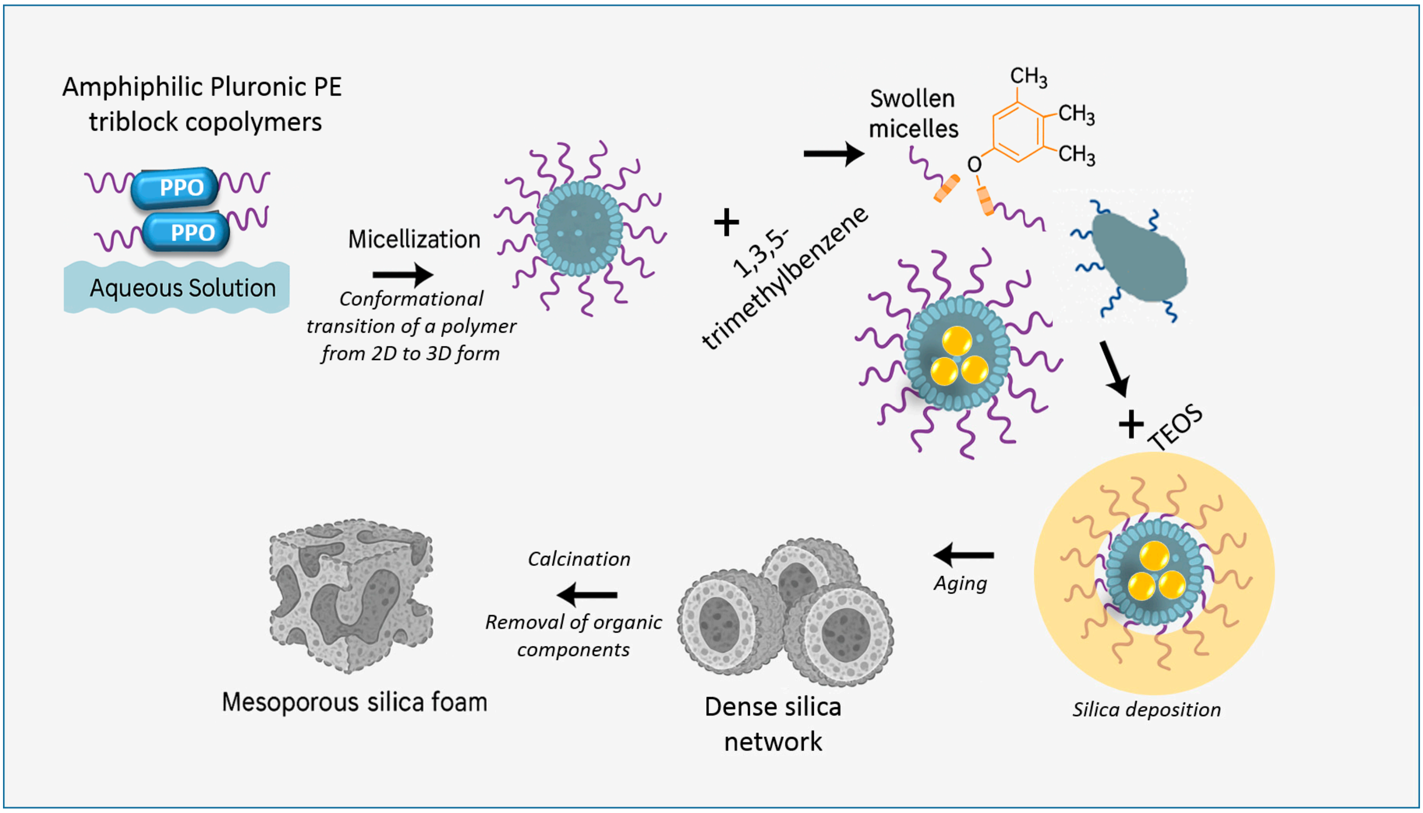

Figure S2. Schema of the MCF silica formation process.

Table S1. Physicochemical properties of the triblock copolymers used in the synthesis of MCF-type materials.

Pluronic trade name	Chemical formula	Molecular weight [g/mol]	Molecular weight of the PO block	Share of EO blocks in the molecule [%]
PE9200	$(EO)_8(PO)_{47}(EO)_8$	3650	2750	20
PE9400	$(EO)_{21}(PO)_{47}(EO)_{21}$	4600	2750	40
PE10500	$(EO)_{37}(PO)_{56}(EO)_{37}$	6500	3250	50
PE6800	$(EO)_{72}(PO)_{28}(EO)_{72}$	8000	1750	80

Table S2. Kinetic models used to optimize the measured data.

No	Kinetic Model	General Equation	Half-Time Expression
1	Multi-exponential (m-exp)	$c = (c_o - c_{eq})\sum_{i=1}^{n} f_i exp(-k_i t) + c_{eq}$ where: c_{eq} and c_0 are the equilibrium and initial concentration, c is the temporary concentration, k_i is the kinetic rate coefficient, t is time, n is the number of exponential terms, coefficient f_i (i=1,2..n) determines fraction of adsorbed equilibrium amount a_{eq} ($f_i = a_i/a_{eq}$)corresponding to adsorption process characterized by rate coefficient k_i.	$t_{0.5,i}$ ~(ln 2)/k_i
2	Fractal-like MOE (f-MOE)	$F = \frac{1 - exp(-k_1 t)^p}{1 - f_2 exp(-k_1 t)^p}$ where: p is the fractal parameter, F is the adsorption progress.	$t_{0.5}$~$[\ln(2-f_2)]^{1/p}/k_1$
2a	Fractal-like FOE (f-FOE)	$F = 1 - exp(-k_1 t)^p$	$t_{0.5}$~$[(\ln 2)]^{1/p}/k_1$
2b	Fractal-like SOE (f-SOE)	$F = \frac{(k_2 t)^p}{1 + (k_2 t)^p}$	$t_{0.5}$~$1/k_2$
